# Hybrid model for analysis of abnormalities in diabetic cardiomyopathy and diabetic retinopathy related images

**DOI:** 10.1186/s40064-016-2152-2

**Published:** 2016-04-23

**Authors:** Fahimuddin Shaik, Anil Kumar Sharma, Syed Musthak Ahmed

**Affiliations:** Electronics and Communication Engineering, SunRise University, Alwar, Rajasthan India; Institute of Engineering and Technology, Alwar, Rajasthan India; Department of ECE, SREC, Warangal, Telangana State India

**Keywords:** Image, Segmentation, Artery, Morphological, Watershed

## Abstract

At present image processing methods hold a noteworthy position in unravelling various medical imaging challenges. The high risk disorders such as diabetic cardiomyopathy and diabetic retinopathy are considered as applications for proposed method. The dictum of this paper is on observing enhancement and segmentation of the cross sectional view of a blood capillary of a right coronary artery image of a diabetic patient and also retinal images. A hybrid model using hybrid morphological reconstruction technique as pre-processing with watershed segmentation method as post-processing is developed in this work.

## Background

Diabetic mellitus (DM) is a metabolic disorder that characterized by incapability of the pancreas to control blood glucose concentration. This dilemma results may make out blood glucose levels out of range (Sharifi et al. 2008). Heart ailments are to blame for 80 % of deaths. Nonetheless, there is an increase in recognition that diabetic patients have a medical condition from an additional cardiac insult termed diabetic cardiomyopathy (Hayat and Patel 2004). Diabetic cardiomyopathy is a situation in which an artery wall thickens as the result of an accumulation of fatty materials such as cholesterol.

The leading pathology in diabetic patients is thickening of basement membrane of a blood vessel making blood vessel narrower leading to occlusion of lumen with fatty materials (Kumar and Shaik 2015). Epidemiological and clinical trial data have recognized the larger incidence and prevalence of heart attacks in diabetes even without a noise at all (Asghar et al. 2009) declaring this as a silent killer.

On the other hand diabetic retinopathy deals with the eye problems which results in visual impairment. This disorder persists for a longer period and an utmost care has to be considered to keep diabetes in control to avoid further consequences.

On a technological note image processing is utilized to extract important features from the images, through which better perception of the scene can be obtained for human viewers (Gonzalez and Woods 1992). The biological vision system is one of the most important means of exploration of the world to humans, making complex task easier for betterment of understanding (Peres et al. 2010). There are numerous algorithms that can be utilised for different applications but enhancement and segmentation are considered as most sort out methods for improving the details in an image. It is not possible to judge that any one method is best in Image processing applications but one can use trial and error method as a practical approach for obtaining the perfect results. Image enhancement is a fundamental task in digital image processing and analysis, aiming to improve the appearance of image in terms of human brightness perception (Intajag et al. 2009). Whereas the segmentation is mainly useful in classification of objects and labelling of the features extracted from image for easy analysis. One should look into that processing of images is done without blemishing the integrity of original image.

## Hybrid morphological reconstruction

Due to the imperfection and variations, the appearance of microscopic images is generally not homogeneous. In order to reduce the influence from undesirable variations within, the hybrid morphological reconstruction (HMR) is used to enhance the image. The steps of HMR are described below.

### Complement of the image

Since the image regions appear darker, we first calculate the complement of the image R, assuming an 8-bit image, as follows:

$${\bar{\text{R}}}({\text{x}},{\text{y}}) = 255 - {\text{R}}({\text{x}},{\text{y}})$$

where (x, y) is the coordinate.

### Opening-by-reconstruction

In order to enhance the regions, the opening-by-reconstruction operation is performed on the image R as follows:

1 $$\bar{R}_{obr} = {\text{R}}\left( {\bar{R}_{e} ,\bar{R}} \right)$$

where R is the morphological reconstruction operator, $\bar{R}_{e} = {\bar{\text{R}}}$ $\ominus$ S ($\ominus$ is the erosion operator), S is the structure element.

### Closing-by-reconstruction

In order to reduce the noise further, the closing-by-reconstruction is performed on *R*_obr_ as follows:

2 $$\bar{R}_{obrcbr} = 255 - \Re (R_{obr} \,{\ominus}\,S,R_{obr} )$$

where *R*_*obr*_ = 255 − $$\bar{R}_{obr}$$

### Complement of the image

This step calculates the complement of $\bar{R}_{obrcbr}$ in order to map the image into the original intensity space i.e.,

3 $${\bar{\text{R}}}^{\prime } (x,y) = 255 - \bar{R}_{obrcbr} (x,y)$$

## Watershed segmentation

**Fig. 1:**
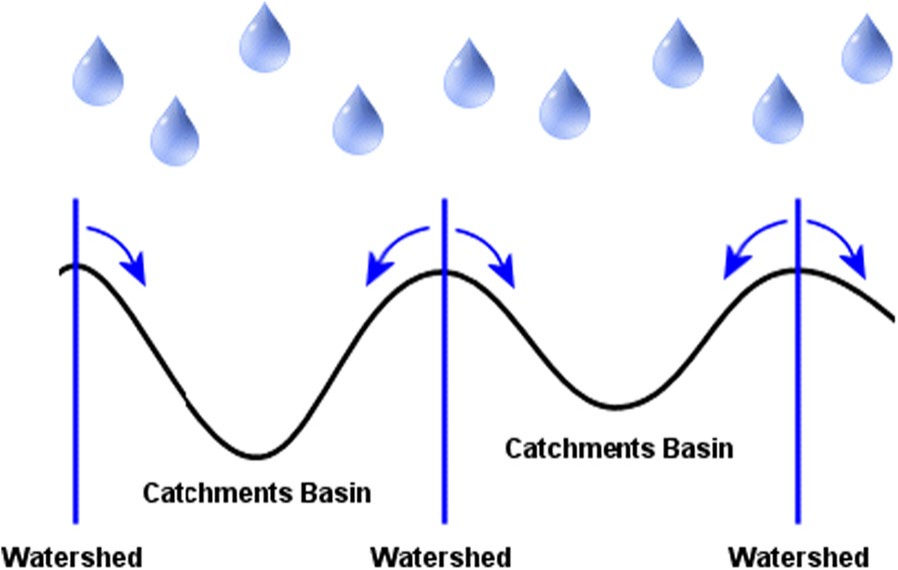
Watershed representation

Watershed segmentation falls under Morphological image processing methods. Morphological operators have been applied for vasculature segmentation (Zana and Klein 1999) because the fundamental morphology of the vasculature is known a priori to be comprised of linked linear segments and because of speed and noise resistance. The concept of watersheds is based on visualizing an image in three dimensions given by two spatial co-ordinates versus intensity. Here one can consider only three points for clear explanation of the topic and they are (a) points belonging to regional minimum (b) points at which a drop of water, if placed at the location of any of those points, would fall with indeed to a single minimum; and (c) points at which water would be equally likely to fall to more than one such minimum. The points fulfilling condition (c) form crest lines on the topographic surface and are termed *divide lines* or *watershed lines* (Shaik et al. 2010) as shown in Fig. 1.

The typical objective of this method is based on the concept to find watershed lines. The basic idea is simple, suppose that a hole is punched in each regional minimum and that the entire geography is flooded from below by allowing water rise through the holes at uniform rate (Ravindraiah and Shaik 2010). The entire process is described by a concept that a dam like thing is constructed to avoid merging and flooding may take place when water reaches the top level of dam. Consequently, watershed algorithm extracts the boundaries. In Tang et al. (2011) watershed algorithm was used for segmentation of splats, a collection of pixels with similar color and spatial location.

## Existing work related to implemented algorithms

In literature (Sopharak et al. 2008; Lu et al. 2012) the above said algorithms have been used as pre-processing or post-processing methods with other algorithms. In Lu et al. (2012) authors used HMR as a pre-processing (mainly to reduce the intensity variation within the nuclei regions and suppress the noise in the image) combined with local region adaptive threshold selection (LRATS) as a post-processing method, based on local optimal threshold to segment the nuclei. In the same paper the researchers used watershed methods separately and compared with their proposed method which is combination of HMR and LRATS. They have concluded that their proposed model is good for attaining the segmentation of nuclei where main images belong to microscopic medical imaging modality.

But the work in Lu et al. (2012) has been not concentrated on combining HMR and watershed methods. Fortunately both of these methods belong to mathematical morphology (here as morphological image processing). A small modification in watershed is done by adding gradient operator into it as an auxiliary function. Thus in this work HMR and modified watershed algorithms are combined to form an improved hybrid model to attain effective results for easy classification and analysis of the inner lying cause of the anomalies present in the images related to diabetes.

## Proposed method

**Fig. 2:**
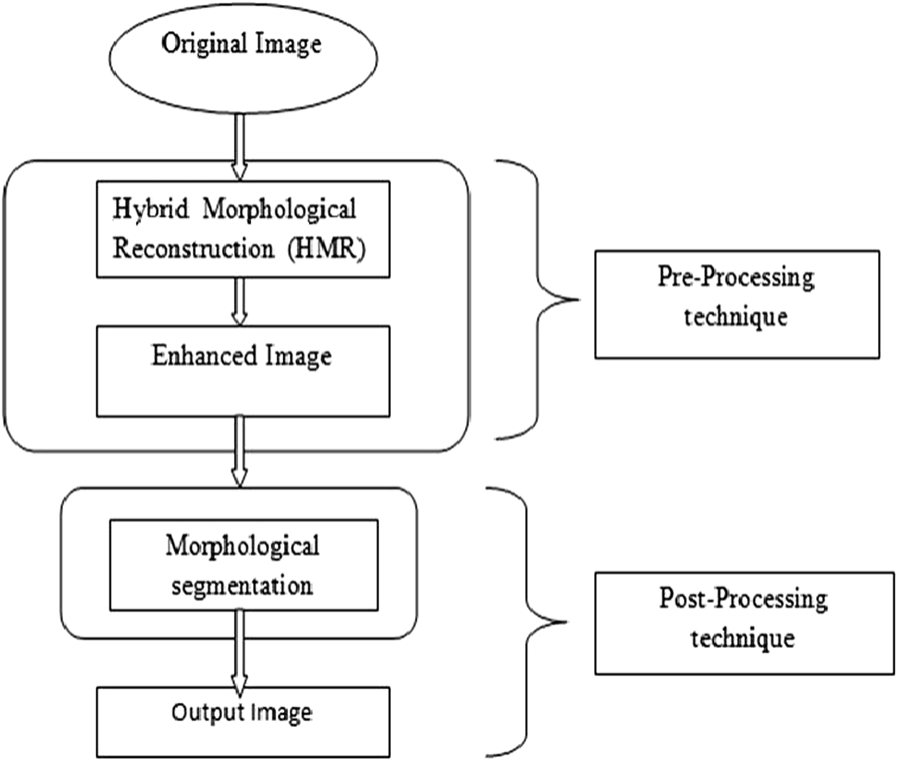
Block diagram of proposed model

The proposed method shown in Fig. 2 is used to enhance and segment the medical images which were considered in this work. Here HMR technique is used to enhance the medical images, and morphological segmentation for classification of the enhanced medical images.

## Discussion on the proposed method

The images that are obtained from the public image database which are related to diabetes mellitus are considered as the input images. The images chiefly taken into work here are of cross section of the right coronary artery (RCA) and retinal images with anomalies. Generally the images are RGB images. So the RGB images are processed using the MATLAB software and the images undergo several algorithms to get a better output. Initially the RGB image is converted into grey scale to avoid complex calculations. Next step is to perform the gradient magnitude segmentation function. After the above two steps are finished then the main step, watershed transform segmentation is performed. Watershed transform is the region base segmentation method. In this step it fills the gaps present in the images and finally the analyzing the result.

## Result and analysis

### Diabetic cardiomyopathy

#### Normal image

**Fig. 3:**
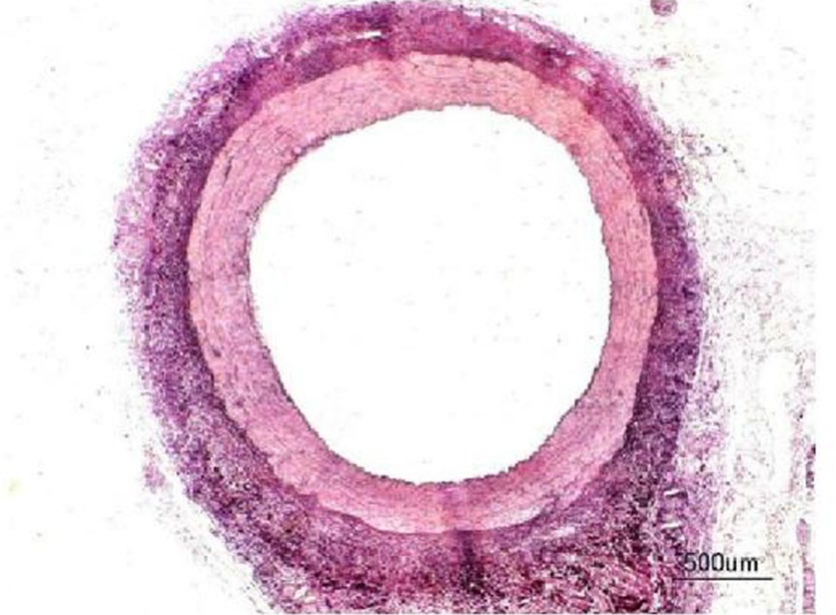
Input image

Figure 3 represents the cross sectional view of a blood capillary of a RCA. Arteries comprise of three layers inter most layer tunica intima, middle layer tunica media and outermost layer tunica adventitia. The artery of the healthy person is considered here.

**Fig. 4:**
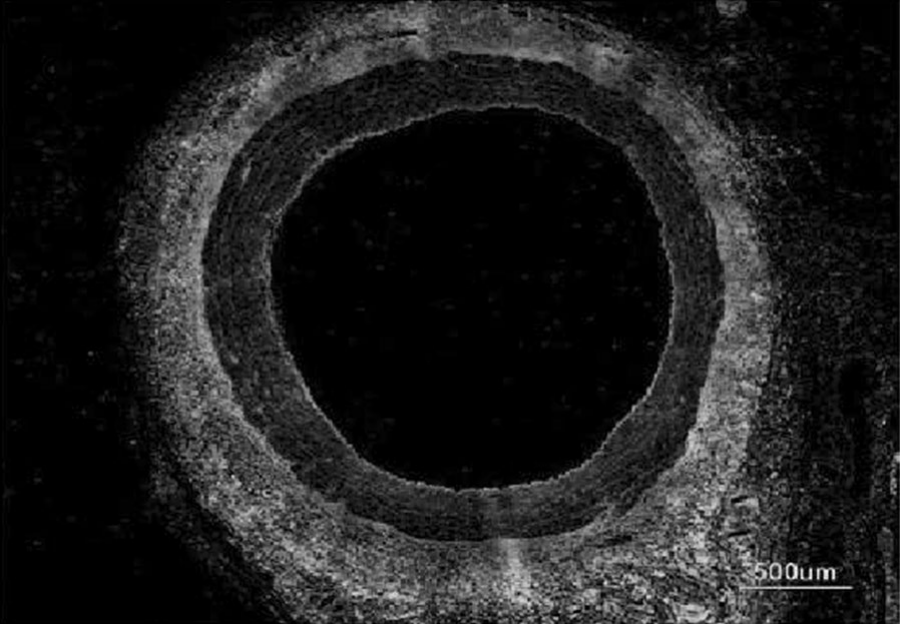
Gray scale image

Figure 4 represents the gray scale image of the original image. Here the pixel values vary from 0 to 255. The operations like top- and bottom-hat cannot be performed straightforwardly on the color images. So in order to execute these pre-processing methods, the RGB image is converted into the gray scale image.

**Fig. 5:**
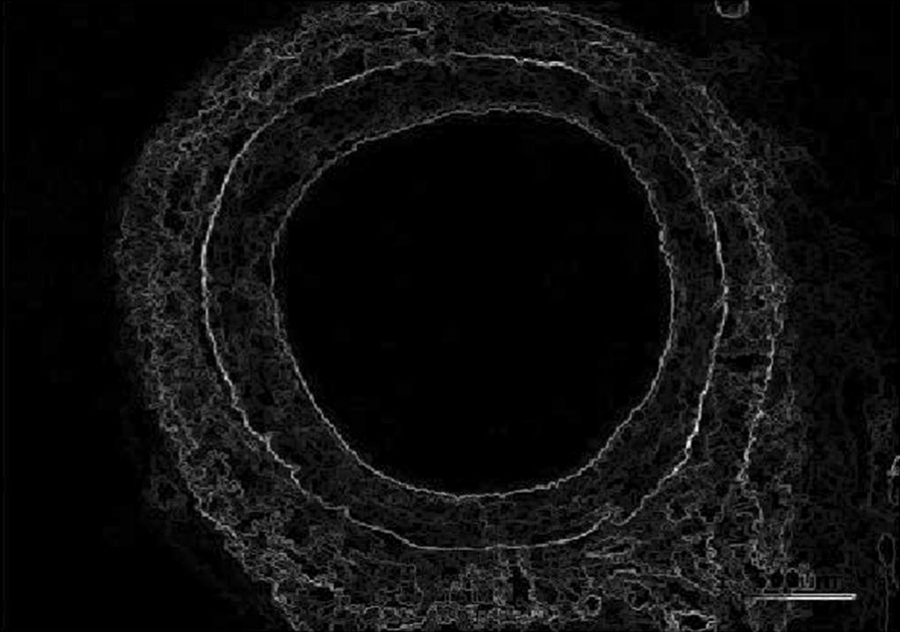
Gradient magnitude image

Sobel filtering is used for the clear detection of edges of the accumulated area. This along with water shed-gradient magnitude gives better results for human perception. Figure 5 clearly gives the forecasting of how lumen will be occluded. Here Sobel edge detection method and a few arithmetic operators are used for better results.

**Fig. 6:**
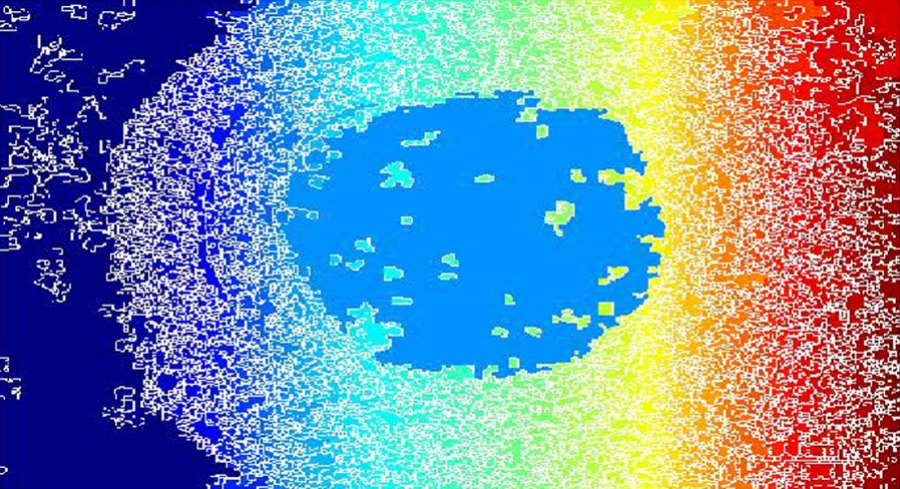
Watershed transform of gradient magnitude

Figure 6 shows the watershed transform of gradient magnitude image. Categorization of touching objects in an image is one of the most complicated image processing operations. In this figure the three layers are obviously seen with lucid boundaries separated.

**Fig. 7:**
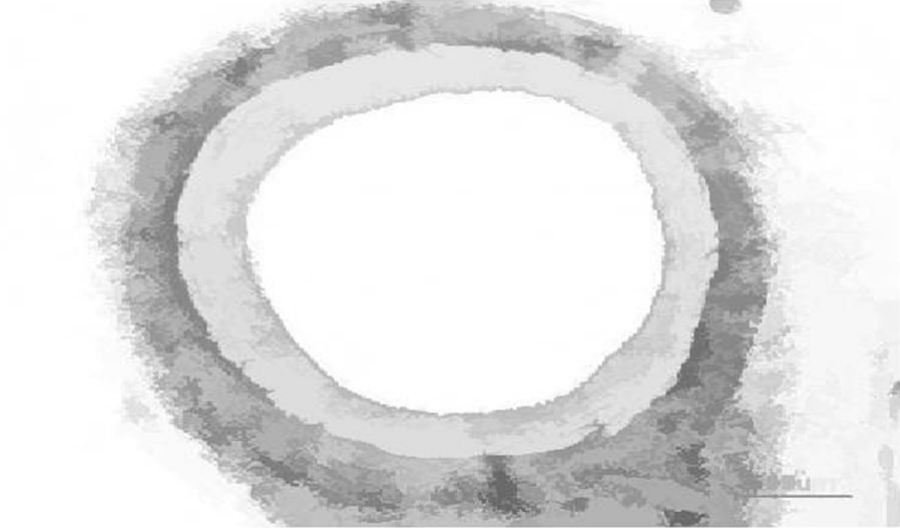
Opening by reconstruction

Figure 7 illustrates the opening by reconstruction image where opening is erosion followed by dilation, here subtraction of pixels is done by erosion process and addition of pixels is done by dilation process. Opening removes small objects from the fore ground of an image. Opening by reconstruction is erosion followed by a morphological reconstruction, where morphological reconstruction is repeated dilations of an image.

**Fig. 8:**
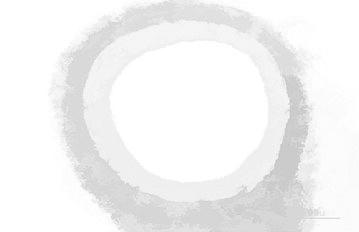
Opening–closing by reconstruction

Figure 8 represents the reconstruction based on opening and closing of the image. The opening with closing can eliminate the dark spots and stem marks. Opening–closing reconstruction based operation is more effective than standard opening and closing at removing small blemishes without affecting the overall shapes of the objects.

**Fig. 9:**
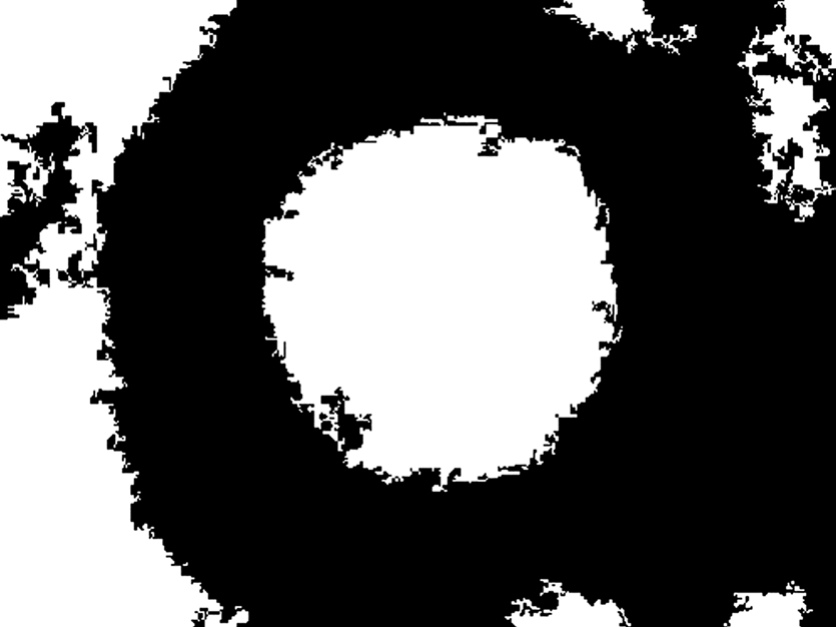
Regional maxima of opening–closing by reconstruction

Figure 9 shows the regional maxima of an opening closing by reconstruction. Regional maxima is a morphological operation which give you an idea about connected components of pixels with a constant intensity value and whose external boundary pixels all have a lower value.

**Fig. 10:**
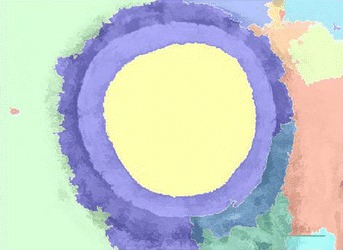
Superimposed image on original image

Figure 10 shows the superimposed image which is useful in demonstrating the division of layers.

#### Medium condition

**Fig. 11:**
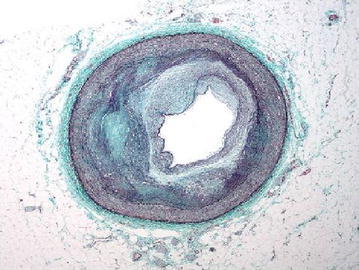
Input image

Figure 11 represents the cross sectional view of a blood capillary of a RCA under the medium condition. Atherosclerosis (is a cardiac insult) leading to narrowing of blood vessel which carries blood to the heart results in heart failure.

**Fig. 12:**
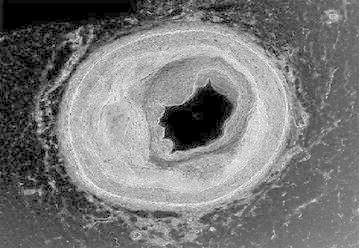
Gray scale image

The operations like top- and bottom-hat cannot be performed directly on the color images. So in order to perform these pre-processing methods, the RGB image is converted into the gray scale image. Figure 12 represents the gray scale image of the original image. Here the pixel values vary from 0 to 255.

**Fig. 13:**
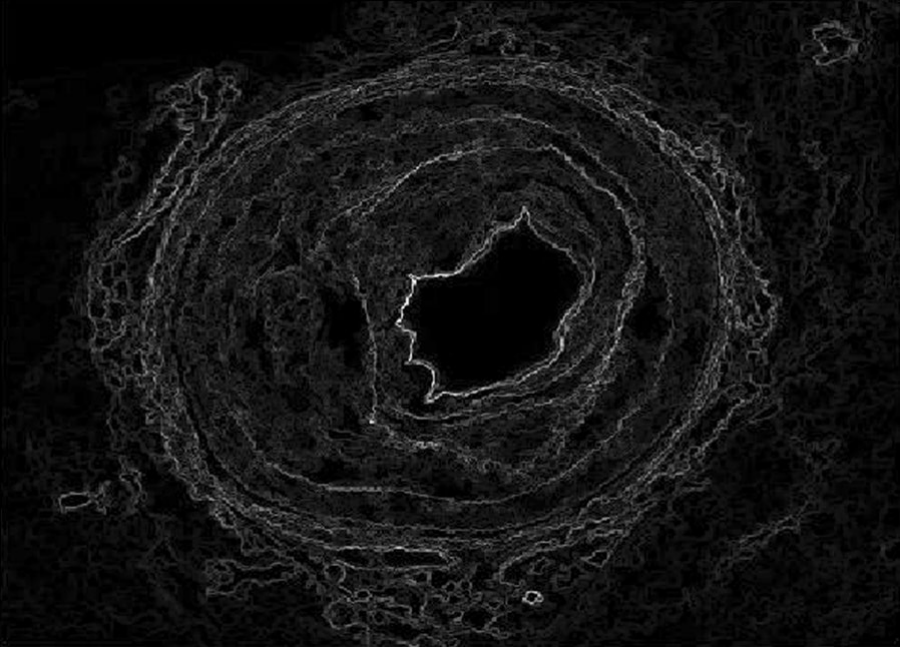
Gradient magnitude

The gradient magnitude image obtained is shown in Fig. 13. For the purpose of edge detection of the region of interest (ROI) (accumulated area) Sobel filtering is used. When it is combined with watershed better results obtained for better perception of underlying abnormality.

**Fig. 14:**
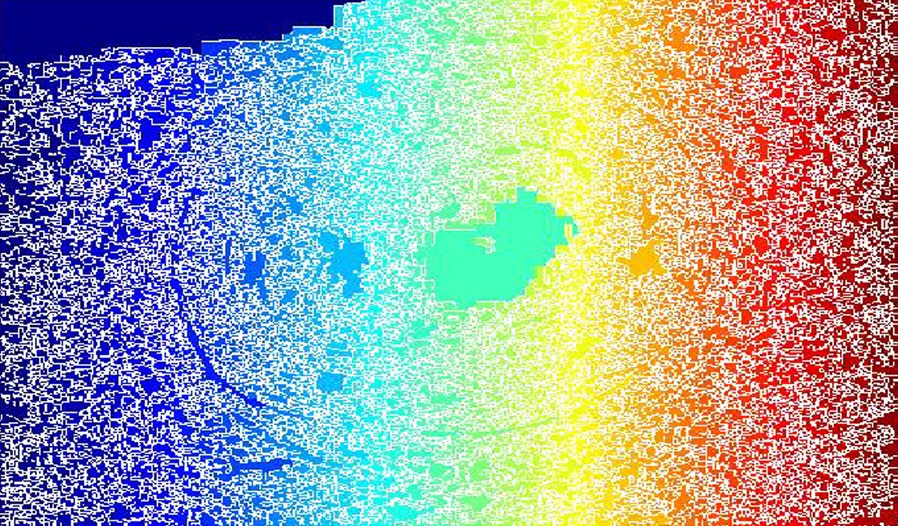
Watershed transform

Watershed transform of the image is shown in Fig. 14 from which one can easily state that the remaining lumen after occlusion.

**Fig. 15:**
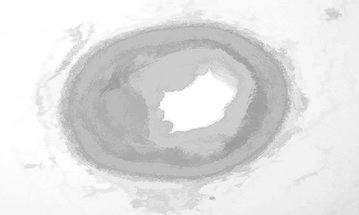
Opening by reconstruction

Opening by reconstruction of the image is shown in Fig. 15. In general opening is erosion followed by dilation, here removal of pixels is done by erosion process and addition of pixels is done by dilation process. Opening removes small objects from the fore ground of an image.

**Fig. 16:**
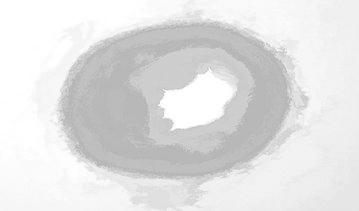
Opening closing by reconstruction

Figure 16 shows the opening closing by reconstruction. The collective operation of opening–closing reconstruction is more effectual than standard opening and closing at eliminating small blemishes without disconcerting the overall shapes of the objects.

**Fig. 17:**
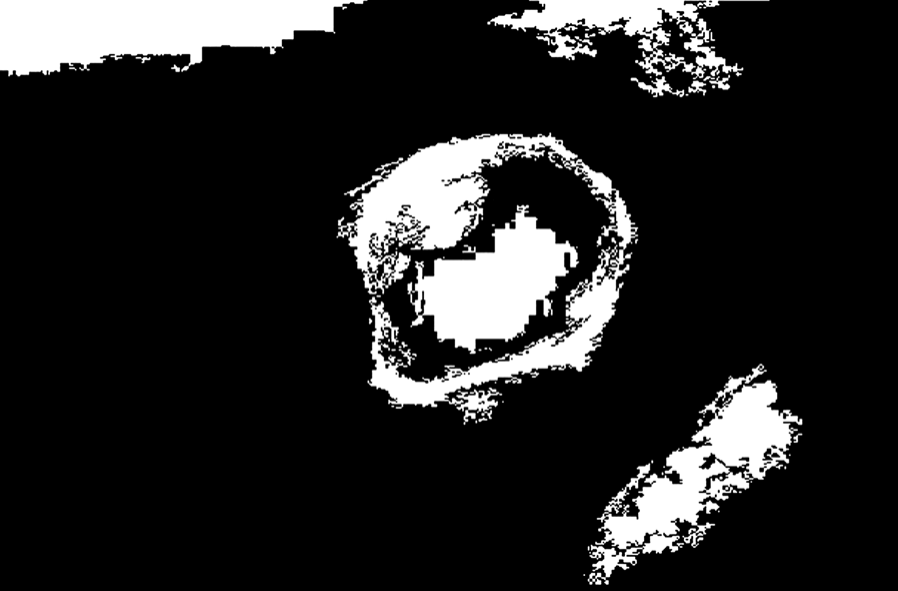
Regional maxima of opening closing by reconstruction

Figure 17 shows the regional maxima of opening closing by reconstruction. Regional maxima is a morphological operation which shows connected components of pixels with a constant intensity value and whose external boundary pixels all have a lower value.

Regional maxima is used to obtain good foreground markers. By applying all these operations we obtained the left area of lumen which is used for the flow of blood. By extending this process, we can alert the common man about the severity and consequences of the disorder if medication is neglected.

**Fig. 18:**
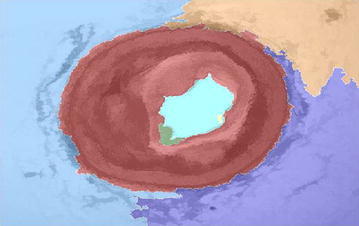
Superimposed image on original image

Figure 18 shows the superimposed image on the original image from which we obtain the problem in the image. This results in comprehensible visualization of the lumen left for blood flow without occlusion.

#### Severe condition

**Fig. 19:**
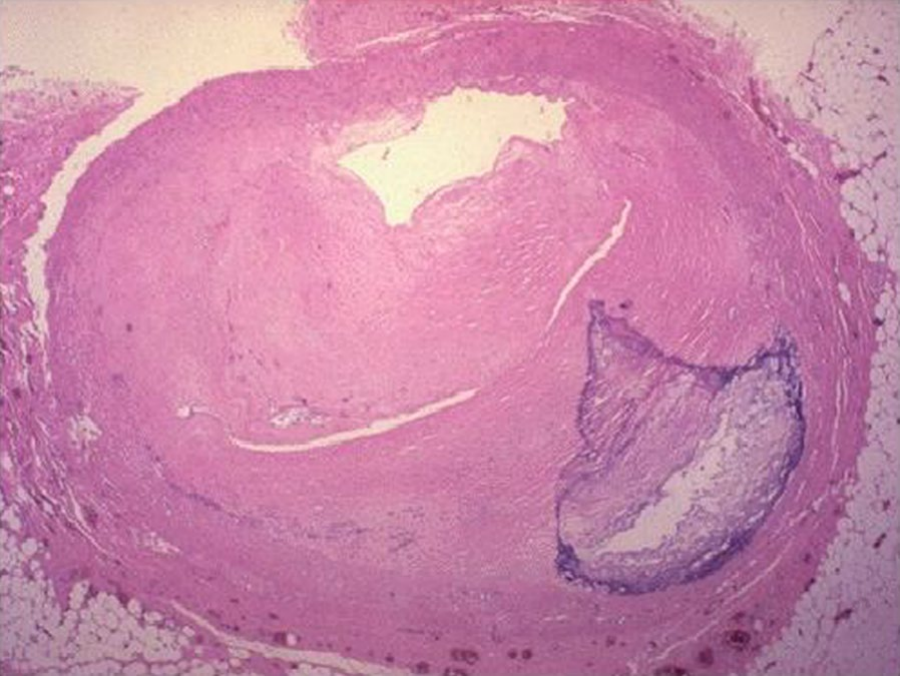
Input image

Figure 19 represents the cross sectional view of a blood capillary of a RCA. The image considered here is of a patient whose artery is almost damaged by the occlusion of lumen.

**Fig. 20:**
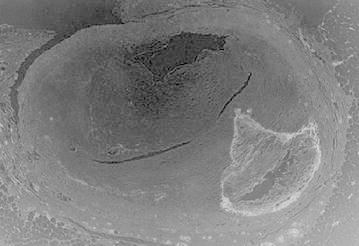
Gray scale image

From above figure it is apparent that the inner most layer i.e., tunica intima is severely coagulated. Figure 20 represents the gray scale image of the original image.

**Fig. 21:**
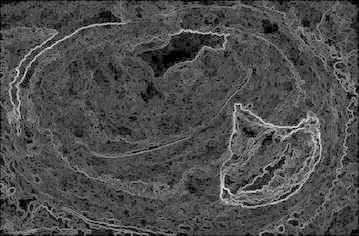
Gradient magnitude image

Figure 21 shows the gradient magnitude of the image. Sobel filtering is used for the clear detection of edges of the accumulated area. This result along with watershed-gradient magnitude gives better results for human observation. The figure clearly gives the forecasting of how lumen will be occluded.

**Fig. 22:**
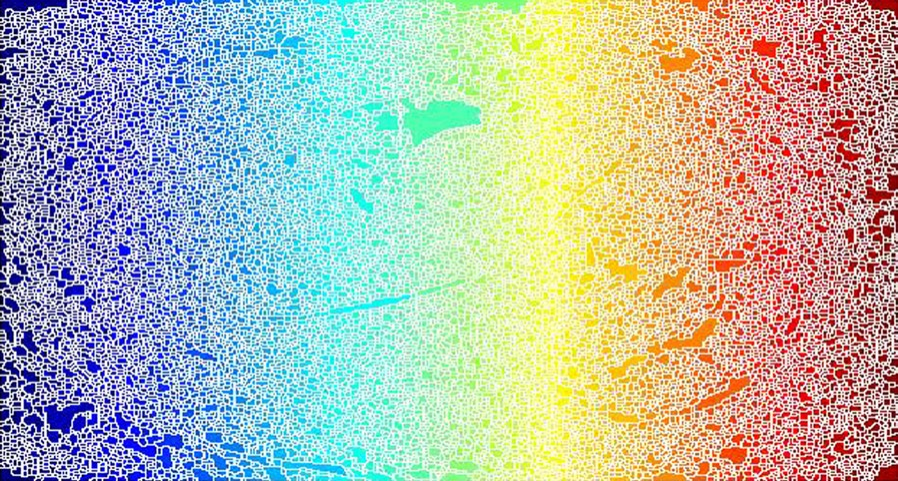
Watershed transform of image

Watershed transform of the image is shown in Fig. 22 from which one can easily state that the remaining lumen after occlusion.

**Fig. 23:**
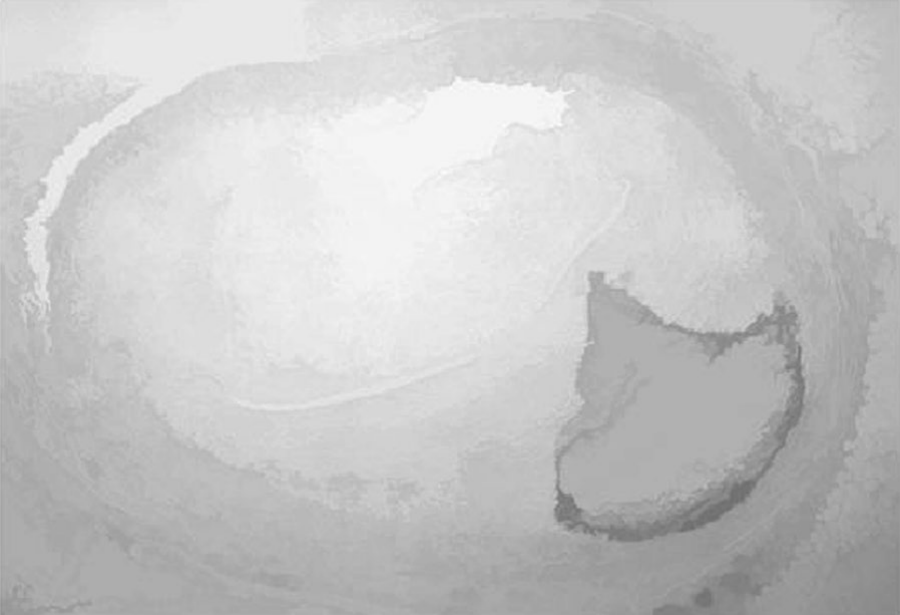
Opening by reconstruction image

Figure 23 shows the opening by reconstruction of the image. Where opening is erosion followed by dilation as discussed in earlier result of Fig. 15.

**Fig. 24:**
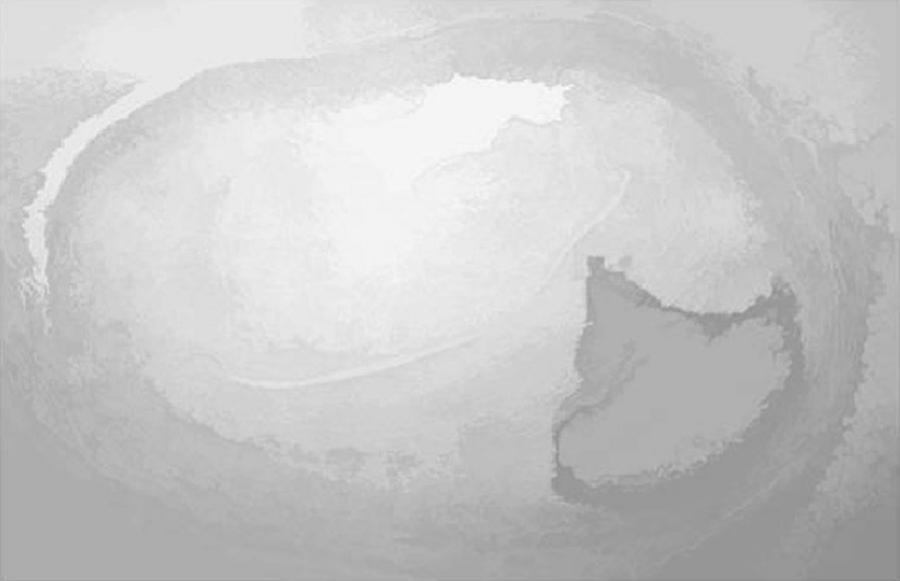
Opening–closing by reconstruction image

Figure 24 represents the reconstruction based on opening and closing image. Opening–closing reconstruction based operation is more effective than standard opening and closing at get rid of small blemishes without upsetting the overall shapes of the objects.

**Fig. 25:**
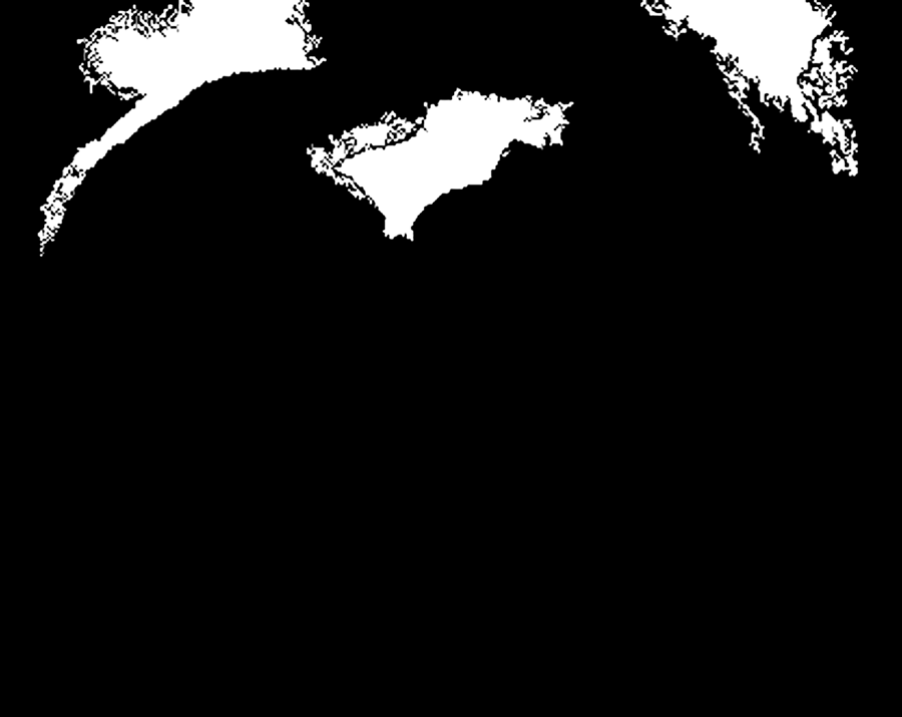
Regional maxima output image

Figure 25 represents the regional maxima of the image. Regional maxima is a morphological operation which shows connected components of pixels with a constant intensity value and whose external boundary pixels all have a lower value. Regional maxima is used to obtain good foreground markers. By applying all these operations we obtained the left area of lumen which is used for the flow of blood. By extending this process, we can alert the common man about the severity and consequences of the disorder if medication is neglected.

**Fig. 26:**
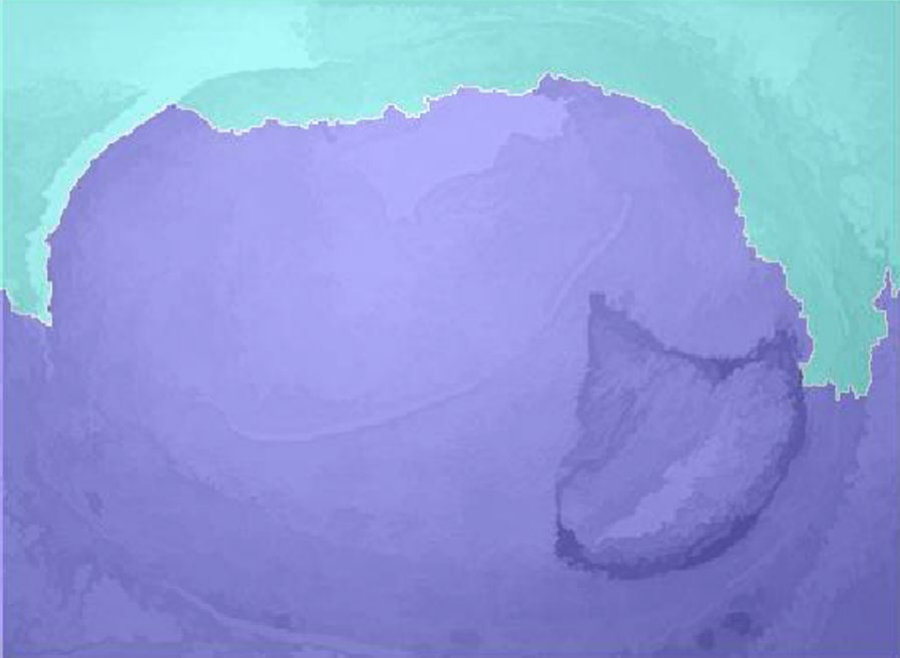
Superimposed on original image

Figure 26 shows the superimposed on the original image from which we can obtain the problem in the image.

### Statistical analysis using MIPAV

Medical image processing, analysis, and visualization (MIPAV) which is an open source is utilized to extract the attributes of significance from the images. These attributes are constructive in elucidation of the abnormalities in precise style. The main feature which makes the researcher to use MIPAV is its applicability even on 3D images and quantification aspect. MIPAV is a Java application and can run on any java enabled PCs. This is the product of Center for Information Technology, National Institutes of Health, Bethesda, MD, USA. The performance of the proposed method was rigorously evaluated using quality metrics like area, perimeter, median, standard deviation of intensity, coefficient of skewness.

By observing area, perimeter and standard deviation of intensity, median, coefficient of skewness the changes occurred in ROI of image after processing are easily revealed.

- Decrease in area indicates that ROI i.e., the degree of severity increases.
- Decrease in perimeter indicates that ROI i.e., the degree of severity increases.
- Increase in the standard deviation indicates that ROI i.e., has been detected with fine edges.
- The variation in skewness (decrease or increase) gives the asymmetry of a distribution.
- Increase in median shows the average changes of the pixels that occurred in the segmented image.

The above all parameters tabulated in Table 1 are obtained by using the medical image processing and visualization (MIPAV) software. By observing the parameters like area and perimeter and then compare the input and out images in each condition i.e., normal, medium, and severe we clearly understand the problem of the images. The calculations are done only to the input and output images but not to each and every image that are obtained in the analysis. For example if we consider the area for three conditions, it will decreased and can be observed from the above table. This means that the gap of the valve is decreased and affects the flow of blood. Perimeter also decreases stage by stage.

**Table 1:** Quality assessment metrics for input and output images (ROI) under normal, medium and normal conditions

Parameters	Normal condition		Medium condition		Severe condition	
	Input image	Output image	Input image	Output image	Input image	Output image
Area	19,971	20,878	5972	6522	3650	3739
Perimeter	576.4831	544.9143	380.627	384.4449	344.3487	350.8232
Standard deviation in RGB	6.9521	5.2928	25.9016	18.8941	6.5016	6.6787
	11.3532	5.2928	24.8035	29.779	15.4811	2.8289
	7.5985	5.2928	20.3622	16.0068	8.8062	6.6281
Skewness in RGB	−5.8341	−4.9663	−4.8262	−3.996	−3.4868	−10.1449
	−5.8166	−4.9663	−4.9189	−4.1371	−2.6438	−6.5082
	−5.6576	−4.9663	−5.24	−3.9746	−1.7176	−5.5639
Median in RGB	196	138	254	253	248	249
	179	214	254	254	234	250
	172	214	254	178	205	174

By tabulating and analyzing these parameters normal people can be alerted about the severity of the problem in three stages i.e., normal, medium and severe conditions.

#### Diabetic retinopathy

**Fig. 27:**
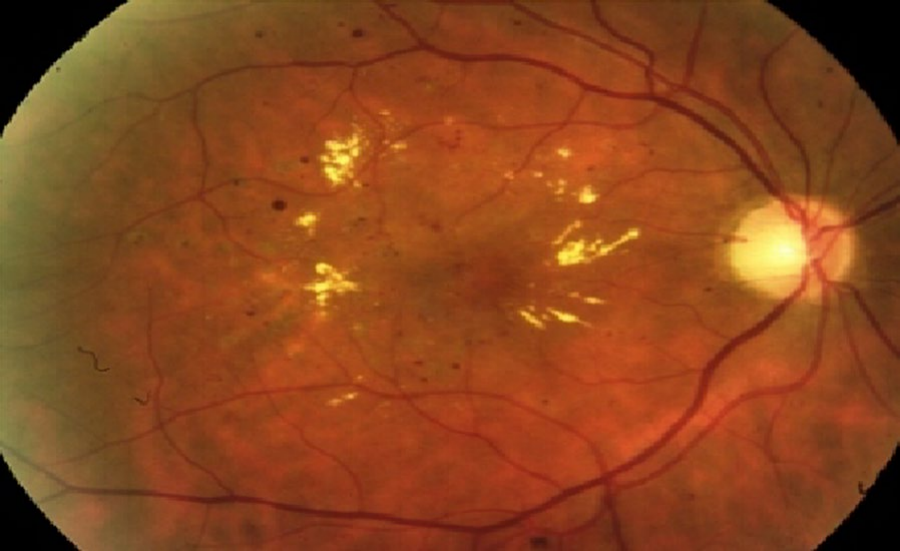
Hard exudates and hemorrhage (courtesy: Illinois Retina & Eye Associates)

**Fig. 28:**
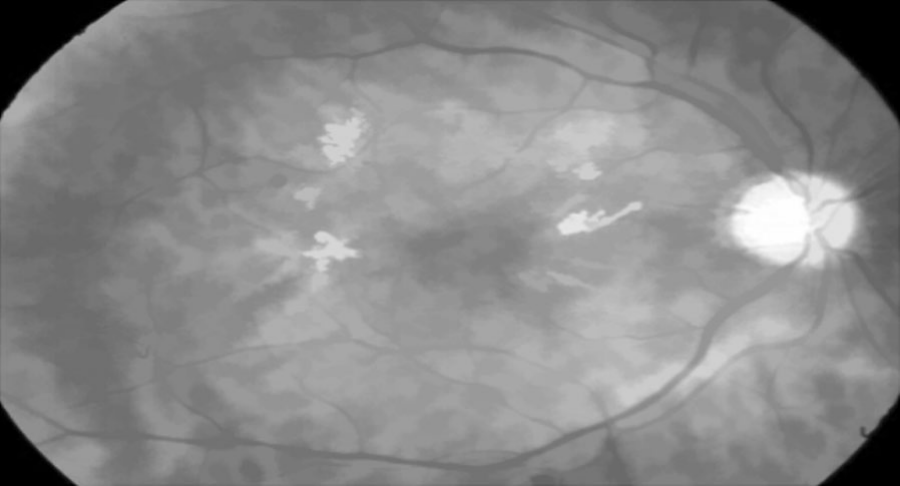
Inverted image

Figure 27 represents DR image with hard exudates and hemorrhages and is fed to the proposed hybrid model. The first stage result is simple inverted image (grayscale image) shown in Fig. 28 yields mainly the hard exudates with a change in the intensity value.

**Fig. 29:**
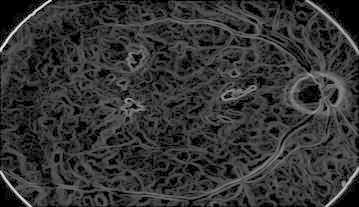
Gradient magnitude

Whereas Fig. 29 is a gradient magnitude image which shows the clear separation of hard exudates from the background represented with arrow mark.

**Fig. 30:**
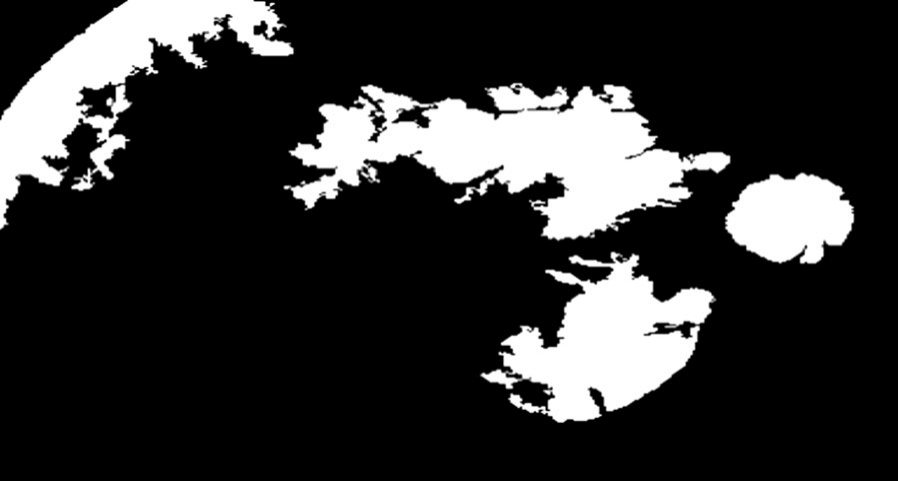
Image reconstruction by regional maxima

**Fig. 31:**
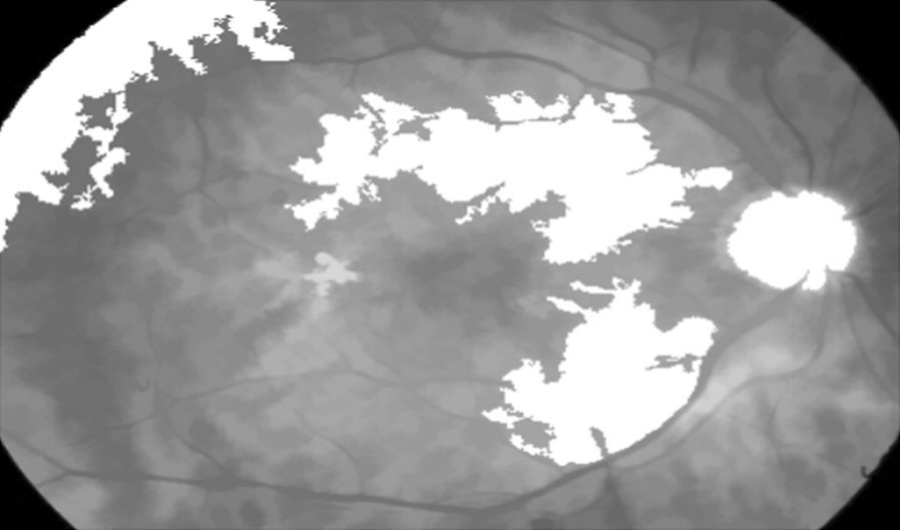
Superimposed image

The resultant images from Figs. 28, 29, 30 and 31 mainly visualize the presence of hemorrhage in the DR image which can be easily perceived from appearance of the images. The output images contain particular ROIs with a change in the intensity value.

## Conclusion and future research

In this study both pre-processing and post processing used belong to the family of Morphological image processing. The experimental results of HMR technique as pre-processing with watershed segmentation method as post-processing are quite suitable for forecasting of narrowing of lumen and retinopathy in diabetic patients. In future development other pre-processing algorithms combined with the implemented post processing method can give perspective results so that the Medical Professionals may make use of this algorithm for earlier detection of the abnormality and this outline in form of a group wise images may be used to alert a common about significance of the problem.
